# Supplementary material for: A set of multi-entry identification keys to African frugivorous flies (Diptera, Tephritidae)
Source: Zookeys. 2014 Jul 24;(428):97–108. doi: 10.3897/zookeys.428.7366 (PMC4143993; doi:10.3897/zookeys.428.7366)
Supplement: Supplementary material 6 — Key to Ceratitis [file zookeys-428-097-s006.zip › SF6_ZooKeys_key to Ceratitis/key/SF6_ZooKeys_key to Ceratitis/Media/Html/Ceratitis lepida .htm]

Ceratitis (Pterandrus) lepida (Munro)


***Ceratitis*** **(*Pterandrus*) *lepida*
(Munro)**

*Pterandrus lepidus* Munro, 1969: 420.

Body
length: 4.81 mm; wing length: 5.25 mm.

Male

Head: Antenna orange-brown. First flagellomere three times
as long as pedicel. Arista missing in holotype. Frons flat, yellow; with short
scattered setulae distinctly darker than frons. Frontal setae missing in
holotype (probably broken). Face yellowish white; gena dark, darkening partly
continued along ventral margin but not in median part; mouthparts black forming
a contrasting character together with dark gena when viewed anteriorly. Genal
seta black; genal setulae black, well developed.

Thorax:
Postpronotal lobe white, without spot. Scutal pattern: ground color dark gray
with silvery shine; with streaks and darker markings but without distinct spots
except prescutellar yellow separate markings. Scapular setae dark. Scutellum
yellowish white, basally without spots, apically with three separate dark spots
extending to halfway scutellar disc. Anepisternum on ventral half black; setulae
on ventral half and across posterior margin black, otherwise pale.

Legs:
Yellow except where otherwise noted; setation typical for subgenus, mainly
dark. Foreleg: coxa black; femur anteriorly with contrasting black/white
pattern, white spot and basal part of femur turn silverly when viewed from
certain angle, anteroventrally with bush of short dark setulae along medial
half; posteriorly dark except apically along ventral side; with dispersed bush
of long dark setulae along entire length, posterodorsal setulae longer; ventral
setae dark; tibia anteriorly mainly brownish black, only distally and along
ventral side partly yellowish. Midleg: femur anteriorly largely brownish black,
only distal end and along dorsal margin partly yellowish; ventrally with dark feathering
along entire length except for small interruption (two-three setae wide) in
median part; tibia broadened and anteriorly largely brownish black, weak
silvery when viewed from certain angle; with black feathering dorsally along
distal 0.9 and ventrally along distal 0.66, second row of short black
feathering present anterior to dorsal feathering, closely apressed to first one
(not easy to differentiate); anteriorly with dispersed short black setulae.
Hindleg: femur brown except apically; at apical 0.25 with longer setulae
dorsally and ventrally.

Wing:
bands yellow and brown. Interruption between marginal and discal bands
near vein R_1_ complete; cubital band free; medial band absent;
crossvein R-M just proximal to middle of discal cell. Apex of vein R_1_
distal to level of crossvein R-M. Crossvein DM-Cu straight. Extension cup
fairly longer.

Abdomen:
Mostly orange-brown. Tergite 1 dark brown across posterior margin. Tergites 2
and 4 with silvery band occupying most if not whole of tergite. Tergite 3 dark
brown posteriorly. Tergite 5 with weak silvery transverse band along� posterior
margin. Male terminalia not dissected.

Female

Unknown.

(Description
after De Meyer & Freidberg, 2006)
